# Biomarkers to predict prognosis and response to checkpoint inhibitors

**DOI:** 10.1007/s10147-017-1122-1

**Published:** 2017-04-05

**Authors:** Takeshi Yuasa, Hitoshi Masuda, Shinya Yamamoto, Noboru Numao, Junji Yonese

**Affiliations:** Department of Urology, Cancer Institute Hospital, Japanese Foundation for Cancer Research, Ariake, Tokyo 135-8550 Japan

**Keywords:** Nivolumab, Biomarker, MSKCC score, IMDC score, Neoantigen, Adverse events

## Abstract

Nivolumab is a fully human immunoglobulin (Ig) G4 antibody that selectively inhibits the programmed death 1 (PD-1) immune checkpoint molecule, and has recently been launched for the treatment of renal cell cancer (RCC) in Japan. Based on its promising anti-tumor efficacy and manageable safety profile demonstrated in the phase III Checkmate 025 trial, nivolumab therapy is rapidly being introduced in metastatic RCC clinical practice. The phase Ia study of atezolizumab, which is a humanized anti-PD-ligand 1 (PD-L1) monoclonal IgG1 antibody, also demonstrated excellent treatment results. The identification of biomarkers to predict the response and side-effects of checkpoint inhibitor therapy is thus urgently needed. In this review, we introduce the current candidate biomarkers of immune checkpoint inhibitor therapy. Based on the mechanism of efficacy, the number of neoantigens and expression of major histocompatibility complex molecules are strong candidate biomarkers. Despite the various interference factors, PD-L1 expression can be considered a potential biomarker. In terms of clinical factors, serum clinical factors and severity of adverse events are examined. Although further implementation in prospective studies is necessary, if validated, these biomarkers can be utilized to measure therapeutic response and design treatment strategies for metastatic RCC.

## Introduction

A greater understanding of molecular biology has led to major breakthroughs in medical treatment for patients with renal cell cancer (RCC). Vascular endothelial growth factor (VEGF), platelet-derived growth factor, and mammalian target of rapamycin (mTOR) signaling pathways have become recognized as rational targets for targeted therapy [1]. Angiogenesis inhibitors, which include sorafenib (Nexavar^®^, Bayer), sunitinib (Sutent^®^, Pfizer), bevacizumab (Avastin^®^, Genentech/Roche), pazopanib (Votrient^®^, Novartis), and axitinib (Inlyta^®^, Pfizer) [2–6]; and two mTOR inhibitors, temsirolimus (Torisel^®^, Pfizer) and everolimus (Afinitor^®^, Novartis) [7, 8], are all currently available as a result of the first breakthrough in metastatic RCC therapy, although bevacizumab has not been approved in Japan.

We are currently on the verge of the second breakthrough. Nivolumab (Opdivo^®^, Ono/Bristol-Myers Squibb) is a novel targeted agent that has just been launched for clinical practice in the treatment of metastatic RCC [9]. Nivolumab, which is a fully human immunoglobulin (Ig) G4 programmed death 1 (PD-1) antibody, selectively inhibits the interaction between PD-1 (which is expressed on activated T cells) and PD-1 ligand 1 (PD-L1) and 2 (PD-L2) (which are expressed on antigen-presenting cells [APCs] and cancer cells) [9]. Its promising anti-tumor efficacy and manageable safety profile were demonstrated in the phase III Checkmate 025 trial. Nivolumab therapy is thus being rapidly introduced in metastatic RCC clinical practice in Japan. Recently, excellent treatment results for the phase Ia study of atezolizumab (Roche/Genentech), which is a humanized anti-PD-L1 monoclonal IgG1 antibody, were also demonstrated [10]. The identification of biomarkers to predict the response and side-effects for checkpoint inhibitor therapy is urgently needed.

Previously, we reviewed the candidate biomarkers of angiogenesis inhibitor therapy in terms of clinical variables, genetic factors, and circulating proteins and endothelial cells [11]. Regarding biomarkers of RCC patients treated with checkpoint inhibitors, however, the role of potential predictive biomarkers to benefit the PD-1/PD-L1 blockade remains controversial and is still under investigation. Most of the ongoing clinical trials have established exploratory biomarker sub-analyses to attempt to identify predictive biomarkers of response to PD-1/PD-L1 inhibition, including PD-L1 expression. Rodriguez-Vida et al. reviewed them comprehensively [12]. Research on other malignancies may also shed light on biomarker analyses in metastatic RCC therapy. Here, we provide a brief overview of biomarkers in terms of the tumor immune microenvironment and clinical factors of RCC and other malignant tumor studies.

## Tumor immune microenvironment

**Fig. 1:**
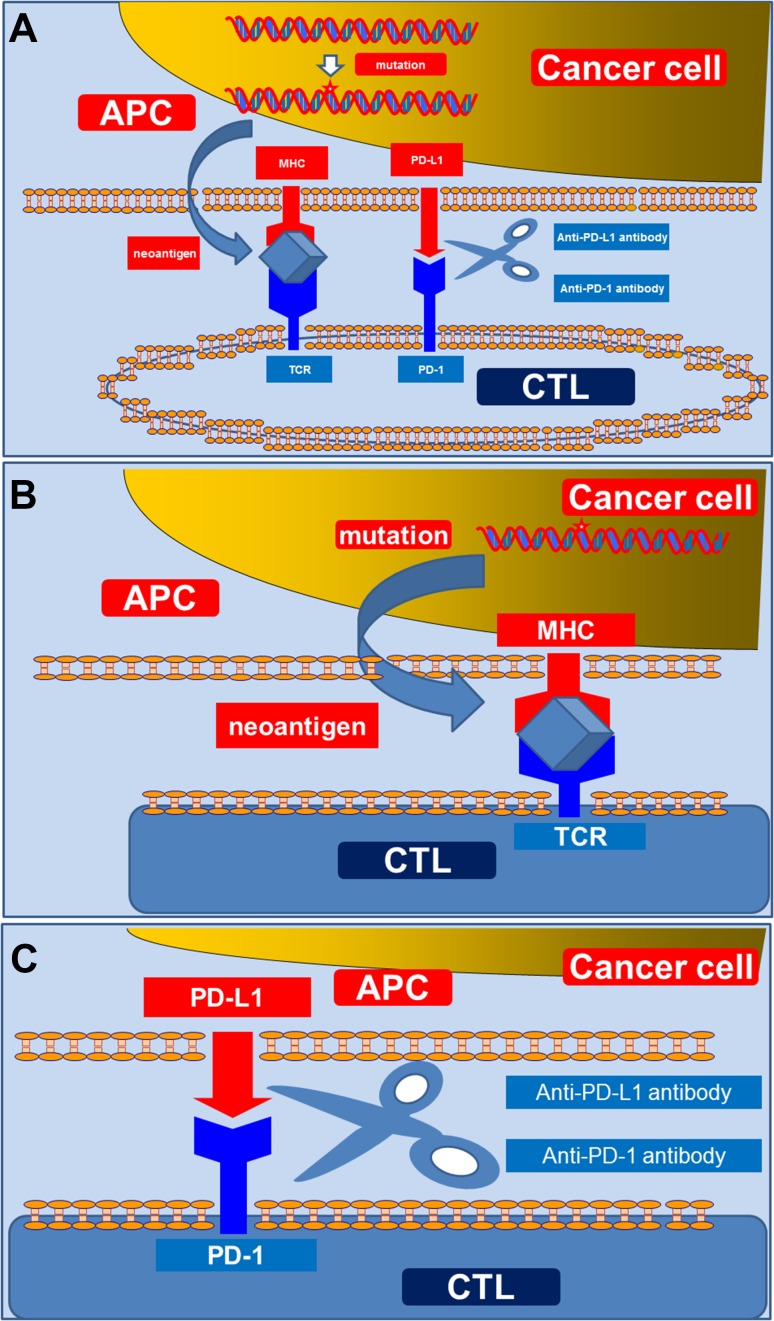
Tumor microenvironment and immune checkpoint inhibitors (Fig. 1a). Cancer cells are recognized by APCs in which cancer cells are processed to peptide antigen; cancer cells are then presented on MHC-I/II as cancer-specific neoantigens. After recognizing these neoantigens, the CTLs are activated and proliferate, and kill neoantigen-bearing cancer cells (Fig. 1b). When a complex of PD-L1 expressed by APCs and cancer cells engage PD-1 expressed on CD8-positive CTLs is complete, immune tolerance is achieved. Destruction of this immune tolerance using immune checkpoint inhibitors is the current novel immune therapy (Fig. 1c). *MHC* major histocompatibility, *CTL* cytotoxic T lymphocytes, *PD*-1 programmed death-1, *PD*-*L*1 programmed death-ligand 1, *APC* antigen-presenting cell, *TCR* T cell receptor

Cancer cells are recognized by APCs in which cancer cells are processed to peptide antigens; cancer cells are then presented on major histocompatibility class I (MHC-I) or class II (MHC-II) molecules as cancer-specific neoantigens [13, 14]. When CD8-positive cytotoxic T lymphocytes (CTLs) recognize these neoantigens presented on the MHC molecules, the CTLs are activated and proliferate, leading to an antigen-specific immune response that kills neoantigen-bearing cancer cells [13, 14]. However, negative regulators exist, namely, the complex of PD-1 and PD-L1/PD-L2. PD-L1 and PD-L2, which are known to be expressed on the surface of APCs and cancer cells, engage PD-1, which expresses on CD8-positive CTLs [13, 14]. When these PD-1 and PD-L1/L2 complexes are complete, they trigger an inhibitory signal to the downstream of the T-cell receptor (TCR), and block effector and CTL functions [13, 14]. Here, immune tolerance is achieved. Destruction of this immune tolerance using immune checkpoint inhibitors forms the basis for the current novel immune therapy (Fig. 1a). In this scenario, the number of neoantigens and the expression of MHC molecules (Fig. 1b) and PD-1/PD-L1 expression (Fig. 1c) can be considered potential biomarkers for immune checkpoint inhibitor therapy.

## Neoantigens and MHC antigens

Neoantigens, which constitute between 8 and 10 peptides, are generally established from tumor-specific mutations, presented by MHC class I or MHC class II molecules on the surface of APCs, and recognized by CD8-positive CTLs that may be able to destroy cancer cells (Fig. 1b) [13, 14]. Although all of the non-synonymous mutations do not always constitute neoantigens, it is probable that the more non-synonymous mutations are affected, the more neoantigens develop. Lawrence et al. investigated the heterogeneity across patients with 27 cancer types, and revealed that the median frequency of non-synonymous mutations varied by >1000-fold across cancer types [15]. Melanoma and lung cancer showed the highest mutation frequencies, exceeding 100/Mb [15]. These may be attributable to extensive exposure to well-known carcinogens, such as ultraviolet radiation in the case of melanoma and tobacco smoke in the case of lung cancers [15]. Among the cancers, RCC (including clear cell cancer and papillary cancer) were in the middle position and demonstrated a low frequency of mutation burden compared with lung cancer and melanoma [15]. Mutation frequencies, however, varied markedly across patients within a cancer type. In clear cell renal cancer, the frequency ranged from 0.1−10/Mb [15]. Rizvi et al. examined the association between the mutation burden and the response of the immune checkpoint inhibitor in non-small cell lung cancer (NSCLC) patients treated with pembrolizumab (Keytruda^®^, MSD), which is a humanized antibody for PD-1 [16]. In this study, they used whole-exome sequencing and reported that higher non-synonymous mutation burden in tumors was associated with improved objective response, durable clinical benefit, and longer progression-free survival (PFS) [16]. In addition, the efficacy was also correlated with the molecular smoking signature, higher neoantigen burden, and DNA repair pathway mutations [16]. Interestingly, although the efficacy was significantly correlated with the molecular smoking signature, self-reported smoking history did not significantly discriminate those most likely to benefit from pembrolizumab [16].

A small fraction of advanced colorectal cancer occurs as a result of mismatch-repair (MMR) deficiency. Uram et al. investigated the efficacy of immune checkpoint inhibitors for colorectal and non-colorectal gastrointestinal cancer patients who have MMR deficiency treated with pembrolizumab [17]. In this study, whole-exome sequencing revealed that a mean of 1782 somatic mutations per tumor in MMR-deficient tumors was much greater than in MMR-proficient tumors, which had a mean of only 73 per tumor (*p* = 0.007) [17]. The median PFS and overall survival (OS) periods, and objective response rate of patients with MMR-deficient colorectal cancer were significantly superior to those of patients with MMR-proficient colorectal cancer [HR for PFS and OS, 0.10 (*p* < 0.001) and 0.22 (*p* = 0.05), respectively] [17]. In addition, patients with MMR-deficient non-colorectal gastrointestinal cancer had responses similar to those of patients with MMR-deficient colorectal cancer [17].

Regarding the receiver of the neoantigen, the expression of MHC antigen might play a role in the efficacy of immune checkpoint inhibitors (Fig. 1b). Using two independent cohorts of anti-PD-1-treated melanoma patients, Johnson et al. reported that MHC-II positivity on cancer cells is associated with therapeutic response, PFS, and OS, as well as CD4 and CD8 tumor infiltration [18]. They concluded that MHC-II expression on cancer cells can be identified by melanoma-specific immunohistochemistry using commercially available antibodies for HLA-DR in order to improve anti-PD-1 patient selection [18]. In addition, in an in vivo study using murine lung cancer cells and anti-mouse PD-1 antibodies, Wang et al. reported that MHC class I and II were significantly downregulated in anti-PD1-resistant tumors compared with anti-PD1-sensitive tumors [19].

## PD-L1 expression

Before describing PD-L1 expression, we must note that there are various factors that influence the PD-L1 expression and clinical efficacy of immune checkpoint inhibitors (Table 1). There are also various assays, including antibodies and cut-off points. There might be a difference between newly collected specimens and archival tumor samples. Furthermore, PD-L1 expression is dynamic and is affected by many factors, including prior therapy and the presence of tumor-infiltrating immune cells, which lead to intra-tumor differences of PD-L1 expression among primary tumors and individual metastatic sites.

**Table 1:** Various interference factors for programmed death-ligand 1 (PD-L1) expression

Used antibody
Immunohistochemistry procedure
Cut-off point of stained sample
Newly corrected specimen or archival tumor sample
Heterogeneity between primary and metastatic sites
Heterogeneity among metastatic sites
Past treatment history

Regarding the difference between PD-L1 expression and the characteristics of RCC, the Mayo Clinic published interesting reports. Thompson et al. reported that PD-L1 expression was demonstrated in both clear cell RCC tumor cells (present in 66% of specimens) and tumor-infiltrating mononuclear cells (present in 59% of specimens) by immunohistochemical analysis [20, 21]. High levels of PD-L1 within the tumors were significantly more likely to exhibit aggressive pathologic features, including higher nuclear grade (*p* < 0.001), positive lymph node metastases (*p* < 0.001), and distant metastases (*p* = 0.022) [20, 21]. In addition, they reported that both metastatic RCC cells and infiltrating lymphocytes express PD-L1 at rates similar to those observed in primary clear cell RCC tumor lesions [20, 21].

Taube et al. investigated PD-L1 and PD-L2 expression of cancer cells and infiltrating immune cells in various cancer types. Cell surface PD-L1 expression by cancer cells and immune-infiltrating cells varied significantly by tumor type, and the most abundant expression was demonstrated in melanoma, NSCLC, and RCC [22]. Expression of PD-L1 by cancer cells and infiltrating immune cells was significantly associated with expression of PD-1 on lymphocytes [22]. PD-L2 expression was also associated with PD-L1 expression [22]. In addition, PD-L1 expression on cancer cells demonstrated a significant correlation with an objective response to anti-PD-1 therapy [22]. Daud et al. also investigated the relationship between anti-PD-1 activity and PD-L1 expression in patients with advanced melanoma who were treated with pembrolizumab in the phase Ib KEYNOTE-001 study [23]. In this study of 451 patients with evaluable PD-L1 expression, 344 (76%) demonstrated PD-L1 expression. High PD-L1 expression demonstrated a significant correlation with a high response rate and long PFS (HR 0.76; 95% confidence interval [CI] 0.71–0.82, *p* < 0.001) and long OS (HR 0.76; 95% CI 0.69–0.83, *p* < 0.001) [23]. Expression of PD-L1 is thus a potential predictive biomarker for response and outcome following treatment with PD-L1/PD-1 immune checkpoint inhibitor therapy.

From the viewpoint of immune checkpoint inhibitor therapy, Hodgkin’s lymphoma is very interesting. In all cases of classical Hodgkin’s lymphoma, Hodgkin Reed−Sternberg cells have copy number alterations of 9p24.1, a region that includes PD-L1 and PD-L2, and contributes to robust expression of these PD-1 ligands [24]. Amplification of 9p24.1 is more common in patients with advanced-stage Hodgkin’s lymphoma. Like a driver gene mutation, amplification of PD-L1 and PD-L2 plays an important role in pathogenesis and treatment resistance in this disease. Before the checkpoint inhibitor therapy era, PD-L1 and PD-L2 amplification was associated with poor prognosis [24]. Therefore, checkpoint inhibitor therapy was warranted. In fact, in the phase I study of nivolumab, 23 patients with relapsed or refractory Hodgkin’s lymphoma, who had already been heavily treated, received nivolumab [25]. An excellent objective response rate of 87% was obtained, including 17% with a complete response and 70% with a partial response [25]. In December 2016 in Japan, nivolumab received approval for treatment of patients with classical Hodgkin’s lymphoma that had relapsed or progressed after initial treatment.

## Clinical factors

In the cytokine era, prognostic factors that could predict outcome in patients with metastatic RCC treated with interferon (IFN)-α as initial systemic therapy were defined by the Memorial Sloan Kettering Cancer Center (MSKCC) study group [26]. The MSKCC group extracted five variable risk factors for short survival—low Karnofsky performance status (PS), high serum lactate dehydrogenase (LDH), low blood hemoglobin (Hb), high corrected serum calcium (Ca), and time from initial RCC diagnosis to start of IFN-α therapy of <1 year (26). Later, the MSKCC group reported the prognostic factors of previously treated RCC patients who had received new agents as salvage therapy [27]. Three factors, including low Karnofsky PS, low Hb level, and high corrected serum Ca level, were extracted as the MSKCC prognostic factors for patients treated by the second-line therapy [27]. In the molecular targeted therapy era, Heng et al. first reported results from a large, multicenter study of 645 patients with anti-VEGF therapy-naive metastatic RCC [28]. In this study, four of the five adverse prognostic factors according to MSKCC score (low Hb, high corrected Ca level, low PS, and time from diagnosis to treatment of <1 year) emerged as independent predictors of poor OS [28]. In addition, high levels of neutrophils and platelets emerged as independent adverse prognostic factors [28]. Later, these prognostic factors were applied to patients previously treated with targeted therapy, in addition to previously validated populations in first-line targeted therapy [29]. These six risk factors are now widely used and are known as the International Metastatic RCC Database Consortium (IMDC) criteria. In the immune checkpoint inhibitor era, these known and widely used criteria must be re-evaluated.

Baseline clinical factors associated with OS after immune checkpoint blockade for melanoma patients treated by pembrolizumab have been reported [30]. Relative eosinophil count ≥1.5%, relative lymphocyte count ≥17.5%, ≤2.5-fold elevation of LDH, and absence of metastasis other than soft tissue/lung were extracted as independent favorable prognostic factors (all *p* < 0.001). In terms of eosinophil count, however, another group reported that eosinophilia was a favorable prognostic factor independent of therapeutic agents [31].

Other groups also reported serum LDH level as a prognostic factor for advanced/metastatic melanoma patients treated with nivolumab or pembrolizumab [32]. After a median follow-up of 9 months, patients with an elevated baseline LDH had a significantly shorter OS compared to patients with normal LDH (6 month OS 60.8 vs 81.6% and 12 month OS 44.2 vs 71.5% (*p* = 0.0292) [32]. In addition, patients with a relative increase of >10% from elevated baseline LDH had a significantly shorter OS compared to patients with a decrease or <10% increase (4.3 vs 15.7 months, *p* = 0.00623) [32]. They concluded that LDH could be a useful marker at baseline as well as during treatment to predict early response or progression in patients with advanced melanoma who received immune checkpoint inhibitor therapy [32]. Similarly, Nakayama et al. reported pretreatment as well as on-treatment prognostic factors for patients with melanoma treated with nivolumab [33]. The Eastern Cooperative Oncology Group (ECOG) PS ≥1, maximum tumor diameter of ≥30 mm, elevated LDH, and elevated C-reactive protein (CRP) were significantly associated with poor OS [HR 0.29 (*p* < 0.001), HR 0.40 (*p* = 0.003), HR 0.29 (*p* < 0.001), HR 0.42 (*p* = 0.004), respectively] on univariate analysis [33]. Among these factors, PS and LDH were identified as independent variables by multivariate analysis [33]. In addition, for early treatment responding markers, patients with absolute lymphocyte count ≥1000/μl [week 3, HR 0.40 (*p* = 0.004); week 6, HR 0.33 (*p* = 0.001)] and absolute neutrophil count <4000/μl [week 3, HR 0.46 (*p* = 0.014); week 6, HR 0.51 (*p* = 0.046)] had significantly better OS [33].

The final topic in terms of clinical factors is adverse events. Are adverse events associated with the efficacy of immune checkpoint inhibitors? In melanoma patients treated with nivolumab, immune-related adverse events (irAEs) are reported to be associated with improved survival [34]. In this study, irAEs of any grade were observed in 68.2% of patients (101 of 148). A statistically significant OS difference was noted among patients with any grade of irAE versus those without (*p* < 0.001), and OS benefit was noted in patients who reported ≥3 irAE events (*p* < 0.001) [34]. In addition, rash and vitiligo correlated with statistically significant OS differences in patients with metastatic disease (*p* = 0.004 and *p* = 0.028, respectively) [34].

## Conclusion

**Fig. 2:**
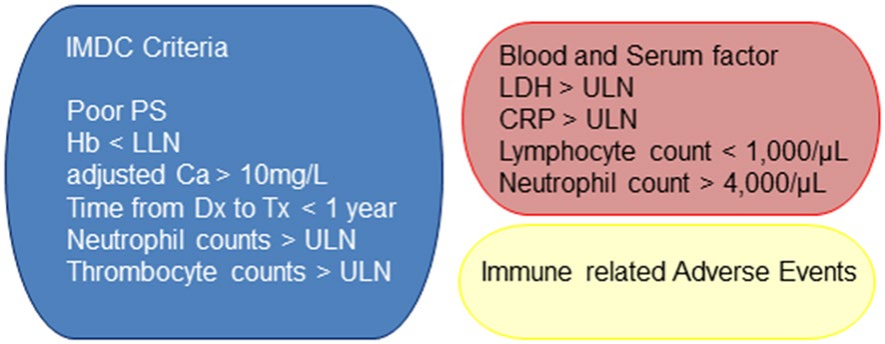
Clinical factors as candidate biomarkers. In addition to various baseline factors, correlations between immune checkpoint inhibitor efficacy and adverse effects have been reported. *PS* performance status, *HB* hemoglobin, *LDH* lactate dehydrogenase, *Ca* calcium, *Dx* diagnosis, *LLN* lower limit of normal range, *ULN* upper limit of normal range, *CRP* C-reactive protein

In this review, we introduced the current candidate biomarkers of immune checkpoint inhibitor therapy. Based on the mechanism of efficacy, the number of neoantigens and expression of MHC molecules are strong candidate biomarkers (Fig. 1b). Despite the various interference factors (Table 1), PD-1/PD-L1 expression can be considered a potential biomarker (Fig. 1c). Regarding clinical factors in metastatic RCC patients, we already have two well-known criteria, including MSKCC and IMDC; however, these widely used criteria must be re-evaluated. Finally, we introduced serum clinical factors and severity of adverse effects as candidate biomarkers of favorable efficacy (Fig. 2). Although further implementation in prospective studies is necessary, if validated, these biomarkers can be utilized to measure therapeutic response and design treatment strategies for metastatic RCC.
